# Tumor stage and treatment outcomes in external auditory canal SCC: a multicenter cohort study

**DOI:** 10.1097/JS9.0000000000004767

**Published:** 2026-04-06

**Authors:** Chung-Wei Lin, Chao-Hui Yang, Yu-Ming Wang, Wei-Chih Chen, Sheng-Dean Luo, Shau-Hsuan Li, Yao-Hsu Yang, Chung-Feng Hwang, Ching-Nung Wu

**Affiliations:** a Department of Otolaryngology, Kaohsiung Chang Gung Memorial Hospital and Chang Gung University College of Medicine, Kaohsiung, Taiwan; b School of Medicine, College of Medicine, Chang Gung University, Taoyuan, Taiwan; c School of Medicine, College of Medicine, National Sun Yat-sen University, Kaohsiung, Taiwan; d Department of Otolaryngology, Kaohsiung Municipal Ta-Tung Hospital, Kaohsiung, Taiwan; e Department of Radiation Oncology, Kaohsiung Chang Gung Memorial Hospital and Chang Gung University College of Medicine, Kaohsiung, Taiwan; f Proton and Radiation Therapy Center, Kaohsiung Chang Gung Memorial Hospital and Chang Gung University College of Medicine, Kaohsiung, Taiwan; g School of Traditional Chinese Medicine, College of Medicine, Chang Gung University, Taoyuan, Taiwan; h College of Medicine, Graduate Institute of Clinical Medical Sciences Chang Gung University, Taoyuan, Taiwan; i Department of Hematology-Oncology, Kaohsiung Chang Gung Memorial Hospital and Chang Gung University College of Medicine, Kaohsiung, Taiwan; j Department of Traditional Chinese Medicine, Chang Gung Memorial Hospital, Chiayi, Taiwan; k Health Information and Epidemiology Laboratory of Chang Gung Memorial Hospital, Chiayi, Taiwan

**Keywords:** external auditory canal carcinoma, squamous cell carcinoma, survival analysis, temporal bone, treatment outcomes

## Abstract

**Background:**

External auditory canal squamous cell carcinoma (EAC SCC) is a rare malignancy with limited evidence guiding treatment strategies. This study sought to evaluate prognostic factors for overall survival (OS), cancer-specific survival (CSS), and time to progression (TTP), and to explore potential differences in outcomes between surgery-based and (chemo)radiotherapy [(C)RT]-based therapies.

**Methods:**

This retrospective multicenter cohort study included 87 patients with histologically confirmed EAC SCC diagnosed between 2007 and 2022 at tertiary hospitals in Taiwan. Patients were grouped by treatment: (1) surgery plus adjuvant (C)RT; (2) (C)RT alone or (C)RT followed by salvage surgery. OS, CSS, and TTP were analyzed using Kaplan–Meier methods, Cox proportional hazards models, and inverse probability of treatment weighting (IPTW). Subgroup analyses were performed to explore treatment-effect modification by age, sex, and tumor stage.

**Results:**

The median age was 66 years, and 64.4% were male. The 5-year OS rate was 46%, with a median follow-up of 2.66 years. T4 tumors (HR 4.98, 95% CI: 1.71–14.5), nodal (HR 3.59, 95% CI: 1.58–8.18), and distant metastasis (HR 2.92, 95% CI: 1.14–7.49) were associated with worse OS, though only tumor stage remained significant after adjustment. Patients treated with (C)RT-based treatment showed poorer OS compared with surgery plus adjuvant (C)RT in crude and IPTW analyses, but these associations attenuated in fully adjusted models. Subgroup analyses suggested that outcome differences were more apparent in older patients, males, and those with advanced tumors although residual confounding effect and the small sample may have influenced these findings.

**Conclusions:**

Tumor stage and treatment modality appeared to be important prognostic factors in this cohort of EAC SCC. While surgery combined with adjuvant (C)RT was associated with more favorable outcomes in several analyses, the observational design limited causal inference. These results should be viewed as hypothesis-generating and underscore the need for larger prospective studies with standardized treatment reporting to clarify optimal strategies.

## Introduction

External auditory canal squamous cell carcinoma (EAC SCC) is a rare malignancy, presenting a diagnostic and therapeutic challenge for clinicians worldwide. With an annual incidence of only 1.3 cases per million^[1]^, it accounts for approximately 0.2% of all head and neck tumors^[2,3]^, underscoring its rarity in head and neck cancer epidemiology. Compared to other histopathological subtypes, such as adenoid cystic carcinoma and basal cell carcinoma, EAC SCC is associated with worse survival outcomes and a higher risk of disease progression^[4–6]^. As the most common malignancy of the external auditory canal^[1,7]^, its low prevalence and poor prognosis pose significant challenges in identifying prognostic factors and optimizing treatment strategies on a global scale.

**HIGHLIGHTS**

- Multicenter cohort study of external auditory canal squamous cell carcinoma over 15 years.
- Advanced tumor stage predicts poor survival and progression.
- Surgery plus adjuvant (chemo)radiotherapy (C)RT] offers better survival than (C)RT-based therapy.
- Older age, male sex, and advanced tumors show stronger treatment disparity.

Surgery remains the standard treatment for resectable EAC SCC^[1,3]^. Common surgical techniques include sleeve resection and temporal bone resection (TBR), which can be performed via en bloc or piecemeal approaches to ensure adequate tumor-free margins^[1]^. Neck dissection, parotidectomy, and adjuvant (chemo)radiotherapy [(C)RT] are often considered for cases with nodal metastases or high recurrence risk^[1,3]^. For unresectable or advanced-stage tumors, definitive radiotherapy (RT) or chemoradiotherapy (CRT) may provide some disease control^[1]^. However, the impact of treatment modalities and clinical characteristics on long-term outcomes, including survival and recurrence, remains unclear. The existing evidence is largely based on small, single-institution series from various countries, which often suffer from limitations, including heterogeneous histopathological subtypes being analyzed collectively and inadequate adjustment for confounding factors^[4,8]^. These methodological gaps highlight the need for further investigation.

The primary objective of this study is to analyze recurrence and survival factors in EAC SCC patients using a multicenter database while accounting for interrelationships among prognostic variables. The secondary objective is to evaluate whether a surgery-based approach confers superior survival and disease control compared to a (C)RT-based treatment strategy.

## Methods

### Data source and study population

The Chang Gung Research Database (CGRD) is a comprehensive, multi-institutional electronic medical records system in Taiwan, derived from Chang Gung Memorial Hospital^[9]^. As the largest database of its kind in Taiwan, the CGRD includes standardized, patient-level data critical for real-world epidemiological research^[10]^. It covers 21.2% of outpatient visits and 12.4% of inpatient admissions nationwide, with even greater representation in specific disease categories. Compared to the National Health Insurance Research Database of Taiwan, the CGRD population demonstrates higher severity of comorbidities and greater disease prevalence. Validation studies indicate a high accuracy of mortality coding in discharge statuses, exceeding 97%, particularly in records after 2010^[11]^. The database’s oncological datasets are further validated through integration with the Taiwan Cancer Registry (TCR), enhancing credibility for epidemiological evaluations.

This retrospective cohort study enrolled patients newly diagnosed with EAC and temporal bone malignancies (ICD-9-CM: 1601, 1732; ICD-10-CM: C301, C442, C4A2) from 1 January 2007 to 30 June 2022, in the CGRD. A total of 109 patients were identified during this period, with diagnoses verified through manual chart review. Exclusion criteria included patients without histopathological confirmation of SCC, resulting in the exclusion of 11 adenoid cystic carcinoma cases, four basal cell carcinoma cases, three adenocarcinoma cases, and four cases with other pathologies. After applying these criteria, the final cohort consisted of 87 eligible patients with EAC SCC. Ethical approval for this study was obtained from the Institutional Review Board of the Kaohsiung branch of CGMH. (Details are available upon request) This study was reported in accordance with the STROCSS 2025 criteria^[12]^.

### Outcome measures and variables

The primary outcomes of this study were overall survival (OS), cancer-specific survival (CSS), and time to progression (TTP). Survival and recurrence data were systematically extracted from the TCR and the CGRD. OS was defined as the time from initial diagnosis to death from any cause, CSS as the time from initial diagnosis to death attributable to EAC SCC or treatment-related complications, and TTP as the time from initial diagnosis to disease progression, including locoregional recurrence or distant metastasis of EAC SCC. Data from the two registries were cross-validated to ensure accuracy and completeness. The last date of follow-up and censoring was 31 December 2023; patients alive without events at this date were censored. Discrepancies were resolved through manual review of clinical records.

A wide range of demographic, clinical, and disease-specific variables were collected to account for potential confounders. Key demographic and clinical factors included patient’s age at diagnosis, gender, body mass index (BMI), smoking history, prior history of head and neck cancers, and comorbidities such as hypertension, diabetes mellitus (DM), chronic kidney disease (CKD) including end-stage renal disease (ESRD), and ischemic heart or brain disease. Disease-specific variables were meticulously recorded, including clinical staging based on the Modified Pittsburgh staging system^[13]^. In this system, T1 tumors are limited to the external auditory canal without bony erosion; T2 tumors exhibit limited bony erosion; T3 tumors extend into the middle ear and/or mastoid; and T4 tumors involve critical structures such as the cochlea, petrous apex, or skull base. Treatment-related factors were also documented, including neoadjuvant chemotherapy, surgical approach, and radiotherapy with or without concurrent chemotherapy [(C)RT].

### Study design and statistical analysis

We first conducted a descriptive assessment to summarize the demographic and clinical characteristics of the study participants. Kaplan–Meier estimators and log-rank tests were used to compare OS, CSS, and TTP across variables of interest. Given that the primary objective of this study was to evaluate the pretreatment clinical characteristics and risk factors associated with oncologic outcomes in EAC SCC patients, aforementioned variables were incorporated to assess in the Cox proportional hazards regression models. Proportional hazards assumption was assessed using Schoenfeld residuals and met for key variables. A primary multivariable model was constructed, controlling for factors with a *P* value < 0.2 in the crude effect analysis. Analyses were performed using complete-case data, with patients missing values for covariates such as BMI or smoking history excluded from the respective multivariable models.

To evaluate oncologic outcomes in EAC SCC, patients were categorized into two groups: intervention group 1, undergoing surgery plus adjuvant (C)RT, and intervention group 2, receiving (C)RT alone or (C)RT followed by salvage surgery. Standardized mean differences (SMD > 0.1) assessed baseline imbalances^[14]^. Propensity scores (PS) were estimated using logistic regression, incorporating age, gender, BMI, smoking history, prior head and neck cancer history, comorbidities, disease-specific factors (location, clinical staging), and receipt of neoadjuvant chemotherapy. To reduce confounding effect, inverse probability of treatment weighting (IPTW) was applied, truncating weights at the 1st and 99th percentiles for stability. These weights were then used in a Cox regression model for survival analysis^[15,16]^. To account for competing risks, subdistribution hazard models (Fine and Gray model) were employed^[17]^. Subgroup analyses explored potential effect modifications across age, gender, tumor status, nodal involvement, and distant metastasis. Sensitivity analyses assessed robustness via^[1]^ incorporating variables with SMD ≥0.1 after IPTW into regression and^[2]^ stratifying PS into quintiles and adjusting for them in a Cox model^[18]^. All statistical analyses were conducted using SAS software (version 9.4, SAS Institute Inc., Cary, NC, USA), with statistical significance set at *P* < 0.05.

## Results

### Patient characteristics

A total of 87 patients with EAC SCC were included. The median age at diagnosis was 66 years (IQR: 56–74), with a male predominance (64.4%). Most tumors originated in the EAC (95.4%), with 4 cases (4.6%) involving the temporal bone. Advanced tumor stages were common, with 57.5% of patients presenting with T3–T4 tumors. Nodal and distant metastases were observed in 9.2% and 5.7% of patients, respectively. Regarding treatment, 24.1% underwent surgery alone, 44.8% received surgery plus adjuvant (C)RT, and 31.0% were treated with (C)RT, either alone or followed by salvage surgery. Additional details on comorbidities, smoking status, prior cancers, and surgical approaches are provided in Table 1. During a median follow-up of 2.66 years (range: 0.3–15.2), 47 patients (54.0%) died from any cause, 24 deaths (27.6%) were cancer-related, and disease progression was observed in 27 patients (31.0%).

**Table 1:** Baseline characteristics and treatment details of patients with squamous cell.

Variables	Patients (n = 87)
Age at diagnosis, median (IQR); years	66 (56–74)
Gender; *n* (%)	
Female	31 (35.6)
Male	56 (64.4)
Body mass index (BMI), median(IQR); kg/m^2^ (n = 81)*	24.5 (22.0-27.7)
Ever-smokers; *n* (%) (n = 72)*	19 (26.4)
History of head and neck cancers	4 (4.6)
Comorbidities; *n* (%)	
Hypertension	23 (26.4)
Diabetes mellitus	19 (21.8)
Chronic kidney disease (including ESRD)	9 (10.3)
Ischemic disease (heart/brain)	13 (14.9)
Location; *n* (%)	
External auditory canal	83 (95.4)
Temporal bone	4 (4.6)
Tumor side; *n* (%)	
Right	48 (55.2)
Left	39 (44.8)
Cancer status	
Tumor status; *n* (%)	
T1	17 (19.5)
T2	20 (23.0)
T3	18 (20.7)
T4	32 (36.8)
Nodal status; *n* (%)	
Negative	79 (90.8)
Positive	8 (9.2)
Metastatic status; *n* (%)	
Absent	82 (94.3)
Present	5 (5.7)
Treatment	
Treatment strategy; *n* (%)	
Surgery alone	21 (24.1)
Surgery plus adjuvant (C)RT	39 (44.8)
(C)RT alone/(C)RT followed by salvage surgery	27 (31.0)
Neoadjuvant chemotherapy; *n* (%)	6 (6.9)
Surgical approach; *n* (%)	
Sleeve resection	11 (17.2)
En bloc temporal bone resection	19 (29.7)
Radical mastoidectomy with piecemeal resection	34 (53.1)
Outcome	
Disease progression; *n* (%)	27 (31.0)
Death; *n* (%)	47 (54.0)
Die of disease; *n* (%)	24 (27.6)

(C)RT, either radiotherapy alone or concurrent chemoradiotherapy; ESRD, end-stage renal disease; IQR, interquartile range.

* Data were missing for BMI (n = 6) and smoking history (n = 15).

### Oncologic outcomes

**Figure 1.**
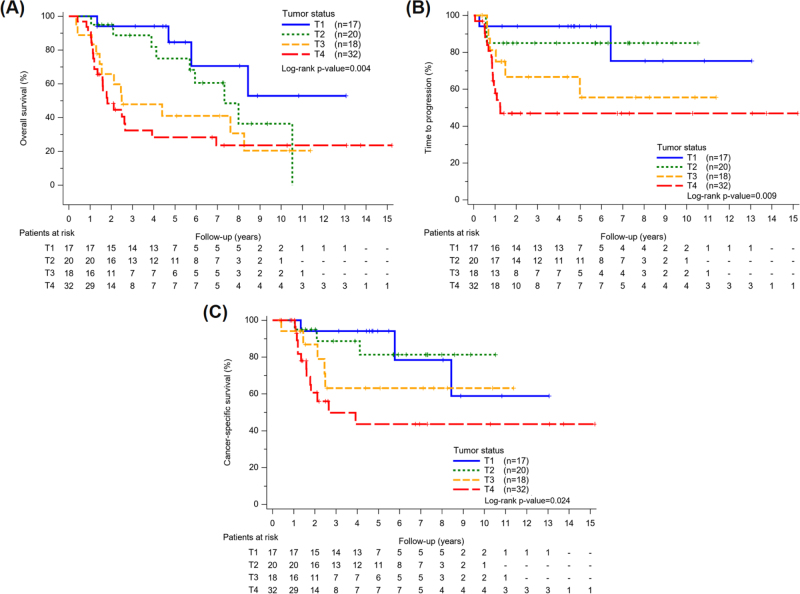
Kaplan–Meier curves for overall survival and time to progression stratified by tumor status. (A) Overall survival (OS) differed significantly across tumor stages (log-rank *P* = 0.004), with worse outcomes in T4 tumors. (B) Time to progression (TTP) also varied (log-rank *P* = 0.009), with faster progression in advanced tumors. (C) Cancer-specific survival (CSS) differed across stages (log-rank *P* = 0.024), with advanced tumors showing worse outcomes.

Crude analysis and Kaplan–Meier curves identified tumor status as a primary prognostic factor for all-cause mortality, disease progression, and cancer-related death (Fig. 1). Compared to T1 tumors, patients with T3 (HR: 4.01, 95% CI: 1.29–12.4) and T4 (HR: 4.98, 95% CI: 1.71–14.5) had significantly higher mortality risks, and also predicted disease progression (T4: HR: 5.78, 95% CI: 1.32–25.2). Advanced T stage was likewise strongly associated with cancer-related death, although effect sizes were attenuated in multivariable models (Table 2). In adjusted analysis, tumor status remained an important prognostic factor, but confidence intervals widened and significance was lost, likely reflecting the limited sample size. Nodal metastasis was initially associated with higher all-cause mortality (HR: 3.59, 95% CI: 1.58–8.18), disease progression (HR: 3.24, 95% CI: 1.22–8.60), and cancer-related death (HR: 5.00, 95% CI: 1.84–13.6), but these associations did not remain statistically significant after adjustment.

**Table 2:** Univariable and multivariable analyses of oncologic outcomes in patients with squamous cell carcinoma of the external auditory canal or temporal bone. Results are presented as hazard ratios (HRs) with 95% confidence intervals (CIs).

Participant characteristics	All-cause mortality (95% CI)		Disease progression (95% CI)		Cancer-related death (95% CI)	
	Crude m00odel	Adjusted model^a^	Crude model	Adjusted model^a^	Crude model	Adjusted model^a^
Age *(per 10-year increase)*	1.08 (0.85–1.36)		0.90 (0.68–1.20)		0.75 (0.55–1.03)	0.82 (0.58–1.16)
Gender (*ref: female*)	0.96 (0.53–1.75)		0.64 (0.30–1.37)		0.68 (0.30–1.52)	
Body mass index (BMI)	1.00 (0.93–1.09)		1.04 (0.96–1.14)		1.08 (0.98–1.19)	*1.11 (1.00–1.23)
Ever-smokers	1.15 (0.56–2.38)		1.17 (0.51–2.68)		1.15 (0.45–2.94)	
History of head and neck cancers	1.15 (0.28–4.76)		N/A		N/A	
Comorbidities						
Hypertension	1.11 (0.58–2.10)		0.76 (0.29–2.01)		0.79 (0.30–2.13)	
Diabetes Mellitus	0.76 (0.37–1.57)		0.67 (0.23–1.93)		0.68 (0.23–2.00)	
CKD (including ESRD)	1.14 (0.45–2.89)		0.37 (0.05–2.73)		N/A	
Ischemic disease (heart/brain)	0.86 (0.36–2.02)		0.73 (0.22–2.42)		0.52 (0.12–2.21)	
Tumor side (*left vs. right*)	1.48 (0.84–2.63)	1.43 (0.76–2.70)	1.78 (0.83–3.80)	1.70 (0.76–3.78)	1.24 (0.56–2.77)	
Location (*temporal bone vs. EAC*)	1.20 (0.29–4.98)		1.84 (0.43–7.77)		1.04 (0.14–7.77)	
Cancer status						
Tumor status (*ref: T1*)						
T2	2.09 (0.64–6.81)	1.58 (0.44–5.73)	1.32 (0.22–7.90)	1.02 (0.16–6.62)	0.93 (0.19–4.60)	1.31 (0.26–6.75)
T3	*4.01 (1.29–12.4)	2.90 (0.89–9.44)	3.46 (0.70–17.1)	2.09 (0.40–10.9)	2.24 (0.53–9.40)	1.41 (0.27–7.29)
T4	*4.98 (1.71–14.5)	3.18 (0.97–10.4)	*5.78 (1.32–25.2)	3.52 (0.71–17.3)	*3.99 (1.13–14.1)	3.81 (0.93–15.6)
Nodal metastasis	*3.59 (1.58–8.18)	1.74 (0.54–5.61)	*3.24 (1.22–8.60)	1.99 (0.71–5.61)	*5.00 (1.84–13.6)	2.52 (0.31–20.7)
Distant metastasis	*2.92 (1.14–7.49)	2.08 (0.54–8.04)	1.79 (0.42–7.58)		3.09 (0.91–10.4)	0.88 (0.08–9.76)
Treatment						
Treatment strategy (*ref: Surgery alone*)						
Surgery plus adjuvant (C)RT	1.47 (0.64–3.36)	1.26 (0.53–3.00)	2.13 (0.59–7.65)	2.14 (0.57–8.06)	0.92 (0.33–2.59)	
(C)RT alone/(C)RT followed by salvage surgery	*3.05 (1.34–6.96)	2.40 (0.97–5.92)	*4.04 (1.15–14.2)	2.98 (0.78–11.3)	1.81 (0.64–5.11)	
Neoadjuvant chemotherapy	1.65 (0.59–4.60)		2.20 (0.66–7.35)	1.99 (0.56–7.05)	1.57 (0.37–6.69)	

CKD, chronic kidney disease; (C)RT, either radiotherapy alone or concurrent chemoradiotherapy; EAC, external auditory canal; ESRD, end-stage renal disease; N/A, not applicable.

^a^ Adjusted model: Adjusted for age and variables with *P* < 0.2 in univariable analysis.

* *P* ≤ 0.05

For treatment effects, crude analysis suggested that patients receiving (C)RT alone or (C)RT followed by salvage surgery had worse outcomes for all-cause mortality and disease progression compared with those who underwent surgery plus adjuvant (C)RT, as reflected in the HR magnitude. This pattern persisted in adjusted models but with wider confidence intervals and loss of statistical significance. When focusing on cancer-related death, the adverse association for the (C)RT-based group was less pronounced than for the other two endpoints, suggesting that part of the observed survival gap may reflect higher non-cancer mortality in this elderly population.

### Treatment comparisons

Before IPTW, patients receiving (C)RT alone or (C)RT followed by salvage surgery (group 2) were older, had lower BMI, and had a higher proportion of advanced tumors (T4: 63.6% vs. 25.8%) compared to those undergoing surgery plus adjuvant (C)RT (group 1), indicating more extensive disease, which was associated with worse survival and disease progression (Supplemental Digital Content Figure S1, available at: http://links.lww.com/JS9/G725). After IPTW, these differences were attenuated but not fully eliminated (Supplemental Digital Content Table S1, available at: http://links.lww.com/JS9/G725). Balance diagnostics are shown in Supplementary Supplemental Digital Content Figures S2–S3, available at: http://links.lww.com/JS9/G725. The Love plot demonstrated substantial reduction in standardized mean differences across most covariates, with residual imbalance persisting for T4 status. Distributional balance plots confirmed improved overlap in covariate distributions between groups after weighting. The propensity score model showed strong discrimination (c-statistic = 0.843). After weighting, the effective sample size decreased from 31 (group 1) vs. 22(group 2) unadjusted to 9.96 vs. 11.71, reflecting efficiency loss due to extreme weights.

Crude analysis showed a significantly higher risk of all-cause mortality in group 2 compared to group 1 (HR: 2.04, 95% CI: 1.09–3.85), which persisted after IPTW adjustment (HR: 2.63, 95% CI: 1.17–5.90) but attenuated and lost statistical significance in regression adjustment and PS stratification (Table 3). These findings were also illustrated in IPTW-adjusted Kaplan–Meier curves, which demonstrated consistently worse survival in the (C)RT-based group (Supplemental Digital Content Figure S4, available at: http://links.lww.com/JS9/G725). For cancer-related death, crude analysis similarly indicated an increased risk in group 2 (HR: 1.95, 95% CI: 0.77–4.93), but this association was not statistically significant and remained insignificant after adjustment, although adjusted estimates consistently remained in above 2. Regarding disease progression, crude analysis suggested a potential increased risk (HR: 1.90, 95% CI: 0.85–4.25), which weakened after IPTW adjustment (HR: 1.21, 95% CI: 0.47–3.13) and remained insignificant across adjusted models. These findings remained consistent after accounting for competing risks.

**Table 3:** Treatment outcomes in patients receiving (C)RT alone or (C)RT followed by salvage surgery vs. surgery plus adjuvant (C)RT.

C)RT alone/(C)RT followed by salvage surgery vs. Surgery plus adjuvant (C)RT (*ref.*)	All-cause mortality			Cancer-related death		Disease progression		
	Cohort number	Death number	Cox proportional hazards model	Death number	Cox proportional hazards model	Progression number	Cause-specific hazards model	Subdistribution hazards model
Crude	66	39	*2.04 (1.09–3.85)	18	1.95 (0.77–4.93)	24	1.90 (0.85–4.25)	1.84 (0.83–4.09)
IPTW	51	30	*2.63 (1.17–5.90)	16	2.48 (0.88–7.02)	22	1.21 (0.47–3.13)	1.13 (0.56–2.25)
IPTW and regression	51	30	2.90 (0.90–9.34)	16	2.41 (0.46–12.6)	22	1.58 (0.44–5.68)	1.69 (0.69–4.18)
PS stratification	53	31	2.05 (0.68–6.24)	16	2.34 (0.53–10.2)	23	1.17 (0.45–3.10)	0.98 (0.39–2.48)

(C)RT, either radiotherapy alone or concurrent chemoradiotherapy; IPTW, inverse probability of treatment weighting; PS, propensity score.

* *P* ≤ 0.05

### Exploratory and sensitivity analyses

Exploratory analyses incorporating clinicopathological and treatment-related variables (Supplemental Digital Content Table S2, available at: http://links.lww.com/JS9/G725) showed no significant associations with OS or TTP across pathological subtypes, likely reflecting small subgroup sizes. Margin status, perineural invasion, and surgical approach did not demonstrate consistent prognostic value, although patients with unassessable margins had significantly higher risk of progression (HR 9.02, 95% CI 1.18–69.07). Facial nerve involvement emerged as a strong adverse factor, with significantly increased risks of both mortality (HR 4.89, 95% CI 2.19–10.94) and progression (HR 5.41, 95% CI 1.80–16.24). Concurrent cisplatin dosing ≥300 mg during radiotherapy was associated with a trend toward improved survival, though the effect did not reach statistical significance. It should be noted that all of these exploratory analyses were based on crude models without adjustment for potential confounders, and thus should be interpreted with caution.

**Figure 2.**
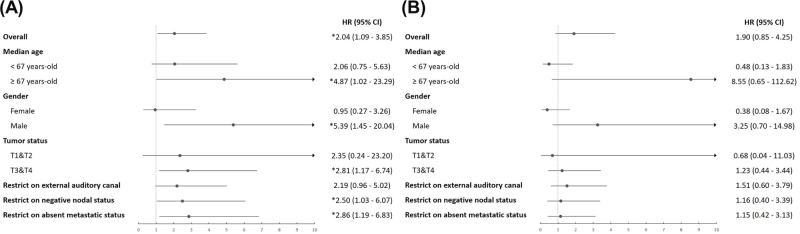
Subgroup analysis of overall survival and time to progression in EAC SCC. Forest plots showing hazard ratios (HRs) and 95% confidence intervals (CIs) for overall survival (A) and time to progression (B), stratified by median age, gender, tumor status, and additional subgroup restrictions. Statistically significant results (*P* < 0.05) are indicated with an asterisk (*). EAC SCC, external auditory canal squamous cell carcinoma

Subgroup analyses suggested that the survival disadvantage of group 2 was more pronounced in patients ≥67 years old, males, and those with T3&T4 tumors, while no significant difference was observed in T1&T2 tumors or female patients (Fig. 2). Sensitivity analyses restricting the cohort to external auditory canal tumors, negative nodal status, and absent metastases showed consistent trends toward worse survival (HRs: 2.19–2.86), though effects on disease progression remained inconclusive (Supplemental Digital Content Table S3, available at: http://links.lww.com/JS9/G725)). Collectively, these exploratory analyses highlight the influence of clinicopathological and treatment-related factors on prognosis, but the limited sample size necessitates cautious interpretation.

## Discussion

This multicenter cohort study evaluated the impact of clinical factors on OS, CSS, and TTP in EAC SCC across a 15-year period. Our findings demonstrate that advanced tumor stage, nodal/distant metastasis, and (C)RT-based treatment are associated with worse oncologic outcomes. Although only tumor stage remained statistically significant after multivariable adjustment, the observed trends suggest that treatment modality and nodal involvement may also play a role in survival and disease progression. Furthermore, our results suggest that surgery plus adjuvant (C)RT may be associated with better survival compared to (C)RT alone or (C)RT followed by salvage surgery; however, this trend was not consistent across all sensitivity analyses and should be interpreted with caution. Subgroup analysis suggested that the survival disadvantage of (C)RT-based treatment appeared more pronounced in older patients (≥67 years), males, and those with T3&T4 tumors, although the limited sample size reduces the robustness of these findings. These findings highlight the need for personalized treatment strategies based on tumor stage and patient characteristics.

To date, studies examining recurrence and survival factors in EAC SCC remain limited and report inconsistent findings. In our cohort, OS rate was 46%, with a median follow-up of 2.66 years, somewhat lower than prior reports ranging from 48% to 70%.^[4,19–22]^ This discrepancy likely reflects our higher proportion of advanced T-stage tumors, which are consistently linked to poorer survival^[22–24]^. Indeed, early-stage cases have demonstrated 5-year OS rates exceeding 90%^[25]^, underscoring the prognostic impact of tumor stage. Our findings reinforce this effect, with T3 and T4 tumors showing elevated mortality risks (T3: HR 4.01; T4: HR 4.98), higher than those reported by Brenet *et al*^[4]^, possibly because our study focused exclusively on SCC, whereas prior studies included heterogeneous histologies^[26]^. Similarly, advanced stage predicted disease progression in our cohort, with T4 stage emerging as an independent predictor. Nodal metastasis also showed associations with OS, TTP, and CSS in univariate models, echoing prior reports^[27,28]^, though significance did not persist after adjustment. Together, these findings highlight tumor stage as the dominant prognostic factor, while nodal status may also contribute to recurrence risk.

In addition to tumor stage and nodal involvement, several pathological and treatment-related variables appeared relevant to prognosis (Supplemental Digital Content Table S2). Facial nerve involvement consistently emerged as a strong adverse factor, aligning with prior studies and reflecting its biological plausibility^[29–31]^. Perineural invasion and close or unassessable margins also trended toward poorer outcomes, underscoring the importance of meticulous pathological assessment. Although higher cumulative cisplatin dose (≥300 mg) suggested improved survival, the small sample size and lack of adjustment for confounders limit firm conclusions. Importantly, radiation dose and field design were not systematically captured in our dataset, which may introduce unmeasured heterogeneity and represent a key limitation. Together, these findings highlight the multifactorial nature of prognosis in EAC SCC and the consequences of heterogeneous treatment practices, including variable use of neoadjuvant chemotherapy and inconsistent radiotherapy reporting. They underscore the need for standardized protocols and collaborative efforts to refine prognostic assessment and optimize treatment strategies in this rare disease setting.

Currently, surgery and (C)RT are the two primary treatment modalities for EAC SCC^[8,32,33]^. Surgery, particularly TBR, is considered the optimal approach for resectable tumors, offering better local disease control and survival outcomes^[6,24,32,34]^. If complete resection is achieved, patients undergoing surgery alone or surgery combined with adjuvant (C)RT generally have improved OS compared to those with unresectable tumors^[8,35,36]^, which are consistent with our findings. When cancer-related death was analyzed as an endpoint, the adverse association for (C)RT-based treatment was less pronounced compared with all-cause mortality and disease progression. This suggests that part of the observed survival disadvantage in the (C)RT-based group may partly reflect higher non-cancer mortality, an important consideration in this comorbidity-prone elderly population. We also acknowledge that patients receiving definitive (C)RT alone and those undergoing salvage surgery after (C)RT failure are biologically and clinically distinct. However, in our cohort, only two patients underwent salvage surgery, precluding meaningful subgroup analysis. To address this issue, we performed a crude sensitivity analysis restricted to patients treated with definitive (C)RT alone (n = 25). Compared with the surgery alone group, this subset demonstrated an increased risk of all-cause mortality (HR: 2.86, 95% CI: 1.24–6.61) and disease progression (HR: 3.66, 95% CI: 1.02–13.15). These results were directionally consistent with the combined (C)RT-based group (n = 27), where crude HRs were 3.05 (95% CI: 1.34–6.96) for mortality and 4.04 (95% CI: 1.15–14.2) for progression (Table 2). The inclusion of the two salvage surgery cases may have contributed to the slightly higher risks in the pooled analysis, suggesting worse outcomes in this subgroup.

The extent to which surgery-based therapy confers a survival advantage requires cautious interpretation due to residual confounding. Although IPTW improved covariate balance across most baseline variables, Love plots demonstrated persistent imbalance in T4 status. The propensity score model achieved good discrimination, but effective sample sizes were reduced after weighting, indicating that extreme weights limited precision. Despite these caveats, the direction and consistency of effect estimates across multiple analytic approaches (HR range: 2.19–2.86) suggest a possible trend toward inferior survival in the (C)RT-based group. This contrasts with findings from Osu *et al*^[37]^ and Ooka *et al*^[38]^, where definitive CRT was reported as non-inferior to surgery in survival outcomes. However, these studies included fewer advanced-stage cases, which may explain the discrepancies in findings. Given the limited literature on the long-term effects of (C)RT-based therapy compared to surgery-based treatment in EAC SCC, further large-scale prospective studies are necessary to clarify the optimal treatment strategy for these patients.

Several limitations should be considered. First, the Modified Pittsburgh staging system’s broad T4 category is a well-documented limitation, as it groups operable and inoperable tumors. This limitation has prompted proposals for alternative systems, such as the T4a/b/c classification by Lavieille *et al*^[4]^. More recently, Berpan *et al* conducted an international comparison of 11 staging systems for carcinoma of the external auditory canal, further underscoring the inconsistencies in prognostic discrimination across classifications^[39]^. Unfortunately, our retrospective dataset lacked the detailed information on anatomical invasion required to apply these more granular systems, making reanalysis unfeasible. We believe that validating superior classification systems in future prospective studies is essential to improving risk stratification. Second, the number of patients experiencing disease progression (TTP) was lower than those with all-cause mortality (OS), which may have reduced statistical power, inflated confidence intervals, and increased the risk of misclassification bias, particularly if recurrences outside the CGRD system were missed. While OS data were reliably obtained through linkage with the National Health Insurance Research Database, ensuring minimal loss to follow-up, underreporting of TTP events may have led to recurrence underestimation and biased risk estimation. In addition, the relatively short median follow-up of 2.66 years, although sufficient for initial survival analyses, may underestimate long-term recurrence or survival outcomes, especially in advanced-stage disease. Third, the relatively small cohort size may have reduced statistical power, particularly in subgroup analyses, which should be interpreted as exploratory and hypothesis-generating. Furthermore, the numerous subgroup and sensitivity analyses raise concerns of type I error, and thus the results should be interpreted with caution. However, as a multicenter study spanning 15 years, this investigation remains among the few to incorporate multivariable adjustments and assess effect modification in EAC SCC. Fourth, as a retrospective study, both treatment selection bias and unmeasured confounders – such as detailed performance status, socioeconomic factors, or patient preference – may have influenced treatment allocation and outcomes. Missing data inherent to registry-based research may also have affected validity. To mitigate potential bias, IPTW was applied to balance baseline characteristics between treatment groups, though residual confounding cannot be entirely excluded. Moreover, potential variations in surgical techniques and radiotherapy protocols across centers may have introduced heterogeneity and influenced outcomes, although the available data did not allow for detailed adjustment. Finally, regarding generalizability, although our data were derived from a multicenter cohort within a single health care system, the study population largely reflects Taiwan’s patient characteristics, given its coverage across multiple regions and focus on advanced cases. Nevertheless, differences in genetic backgrounds, environmental exposures, and treatment practices between Asian and Western populations may limit the direct extrapolation of our findings. Future international multicenter studies with extended follow-up are warranted to validate whether these results are applicable to broader global cohorts.

Our findings may also have implications for clinical practice and research. They suggest that surgery could be considered a favorable option when tumors are resectable, while the interpretation of (C)RT outcomes should take into account potential contributions from comorbidities and non-cancer mortality. For future research, our study highlights the importance of prospective, multicenter collaborations with standardized reporting of surgical margins, radiotherapy parameters, and chemotherapy regimens. Such efforts will be essential to refine prognostic models and to provide more reliable evidence for guiding treatment strategies in this rare disease.

## Conclusion

In this multicenter cohort, advanced tumor stage was the most important predictor of survival in patients with EAC SCC. Surgery-based therapy appeared to be associated with more favorable outcomes in resectable cases; however, these findings should be interpreted cautiously given the retrospective design, limited sample size, and variability across sensitivity analyses, and they warrant confirmation in prospective studies.

## Data Availability

The data used in this study are owned by Chang Gung Medical Branches and cannot be shared publicly. Access to the data is restricted and available only to researchers who meet the necessary criteria for confidential data access. Interested researchers may request access by applying through the Department of Medical Research and Development at Chang Gung Memorial Hospital. For inquiries, please contact Dr. Ching-Nung Wu via email at taytay@cgmh.org.tw.

## Research registration unique identifying number (UIN)

This is a retrospective database analysis that was approved by the institutional IRB, and no UIN registration was applicable.

## AI usage statement

Purpose and scope of AI use

Artificial intelligence (AI) was employed to assist in language drafting, language editing, and refining the clarity and conciseness of the manuscript text. No generative AI was used for data analysis, statistical interpretation, or clinical decision-making. Specifically, AI was utilized during the writing and revision stages to ensure the clarity of the scientific language. The authors take full responsibility for the integrity and accuracy of the content, including any parts refined or generated using AI assistance.

## AI tool(s) and configuration

The AI tool used was OpenAI’s ChatGPT (model: GPT-4, version as of 2025-06-01), accessed via a secure cloud-based API without local deployment. No plug-ins, fine-tuning, or specialized prompt engineering (e.g., temperature or model modification) was applied beyond the default parameters for general academic writing assistance.

## Data inputs and safeguards

No patient data, identifiable images, or sensitive personal information was provided to the AI system. Only de-identified manuscript text drafts were used as inputs. All inputs complied with GDPR/HIPAA standards, and no additional institutional data-sharing approvals were required as no patient-level data was involved.

## Human oversight and verification

All AI-generated or refined text was reviewed by the corresponding author (Dr. Ching-Nung Wu) and senior co-authors. The outputs were fact-checked for clinical accuracy, scientific correctness, and relevance. Any generated text that did not meet academic standards was revised or discarded. The authors acknowledge the limitations of AI, including the risk of misinterpretation, and confirm that all final manuscript content reflects the authors’ expertise and judgment.

## Reproducibility and transparency

Exact prompts and AI interaction logs can be provided as supplementary material upon request for independent review and replication. No permanent or shareable repository was created, as AI usage was limited to internal language refinement.
